# Prevalence and Incidence of Oral Benzodiazepine Use in Hospitalized Surgical Patients: A Retrospective Cohort Study

**DOI:** 10.1097/SLA.0000000000006647

**Published:** 2025-01-30

**Authors:** Roos Geensen, Jorrit G. Verhoeven, Johanna M. Hendriks, Wim J.R. Rietdijk, Johannes Jeekel, Nicole G.M. Hunfeld, Markus Klimek

**Affiliations:** * Department of Neuroscience, Erasmus MC University Medical Centre, Rotterdam, The Netherlands; † Department of Surgery, Erasmus MC University Medical Centre, Rotterdam, The Netherlands; ‡ Department of Hospital Pharmacy, Erasmus MC University Medical Centre, Rotterdam, The Netherlands; § Department of Intensive Care, Erasmus MC University Medical Centre, Rotterdam, The Netherlands; ‖ Department of Anaesthesiology, Erasmus MC University Medical Centre, Rotterdam, The Netherlands

**Keywords:** benzodiazepine, hospitalization, surgical patients

## Abstract

**Objective:**

To determine the prevalence of intrahospital oral benzodiazepine use in the surgical population of a tertiary care centre.

**Background:**

Oral benzodiazepines used for treating sleep disturbances and anxiety are widely used in the general population. Information regarding benzodiazepine use during hospitalization is scarce.

**Methods:**

A retrospective cohort study was conducted using routinely collected health care data in a university hospital in Rotterdam, The Netherlands. In this cohort, 10,896 patients representing 14,928 admissions were included, corresponding to all adult surgical patients admitted between September 2018 and September 2022. Median age was 62 (50–72) and 8761 out of 14928 (58.9%) were males. The main outcome measures were the prevalence and incidence of oral benzodiazepine usage during hospitalization.

**Results:**

Prevalence of benzodiazepine administrations in the surgical department was 21.6% out of 14,928 admissions. Median number of tablets given during hospital stay was 3 (1–7). Temazepam (33%), oxazepam (24%), and zopiclone (19%) were prescribed most. Female patients were more likely to have been administered a benzodiazepine, with an adjusted odds ratio of 1.09 (95% CI: 1.002–1.19). Benzodiazepine administration during admission was positively associated with higher 30-day surgical readmission, with an adjusted odds ratio of 1.37 (1.22–1.54).

**Conclusions:**

In this study, one-fifth of patients admitted to surgical departments were administered oral benzodiazepines for sleep disturbances and anxiety. Future research and policies should focus on finding and implementing effective non-pharmacological methods for perioperative sleep disturbances and anxiety.

Benzodiazepines and benzodiazepine receptor agonists, often referred to as “Z-drugs,” are a subset of hypnotic drugs that are commonly used to treat anxiety and sleep disturbances. Over the last decade, prescription rates of benzodiazepines have been increasing.^1^ However, increased attention to benzodiazepines’ hazardous side effects has made clinicians wary of overprescription and unwanted prolonged use.^1–3^ Long-term benzodiazepine use leads to cognitive dysfunction.^4,5^ Moreover, research suggests benzodiazepine use in hospitalized elderly and critically ill patients leads to an increased risk of delirium.^6,7^ Although recent studies indicate single-dose perioperative benzodiazepines do not increase the risk of postoperative delirium,^8^ nor do single preoperative doses of anxiolytic benzodiazepines known as premedication.^9^ Randomized controlled trials, however, have shown that premedication does not increase the quality of recovery.^10,11^ These and other seemingly contradictory results have led to a renewed pro-con debate in the perioperative care field.^12^ This debate notwithstanding, in general, there has been a movement towards limiting inappropriate use of benzodiazepines and raising awareness for gradual dose reduction and deprescribing, especially in the elderly.^13–15^

As a result, benzodiazepine prescription rates in the general population have followed declining trends in many countries, including The Netherlands.^16–18^ However, in 2021, that trend was broken in the Netherlands as the number of prescriptions of benzodiazepines rose again. This was mainly attributed to a 9.7% increased use of temazepam, a benzodiazepine that is primarily prescribed as a pharmacological treatment of sleep disturbance.^19^ Considering the potential harmful effects of inappropriate benzodiazepine use, epidemiological research has attempted to identify risk factors for new benzodiazepine use, with limited success. One proposed factor is new perioperative use of benzodiazepines, in a similar manner to how persistent opioid usage is often elicited by a surgical procedure and concurrent hospital admission.^20,21^

Data regarding the prevalence of benzodiazepine use during hospitalization, specifically in surgical patients, is scarce and inconsistent. A Swiss retrospective hospital-wide cohort study found that 48.3% of all admitted patients were treated with benzodiazepines at least once.^22^ Focusing on surgical patients only, a large nationwide American cohort study of patients undergoing common surgical procedures found a new perioperative benzodiazepine prescription rate of 2.6%.^23^ A smaller, but similar cohort study from Iceland showed that 2.7% of benzodiazepine naive patients undergoing surgery at a tertiary centre received benzodiazepines prescriptions perioperatively.^20^ For both these studies investigating surgical patients, perioperative benzodiazepine use was defined as use between 30 days before, until 14 days after surgery, and study populations were heterogeneous. In conclusion, information regarding the actual in-hospital benzodiazepine use in the surgical population is absent. Therefore, the objective of this study is to study the prevalence and incidence of intrahospital oral benzodiazepine use in the surgical departments of a university centre.

## METHODS

### Study Characteristics

The Erasmus MC is the largest academic teaching hospital in the Netherlands and functions as a tertiary care provider for the country. Data from the electronic health record (EHR) was extracted by data engineers according to predefined queries. Final data extraction was performed on March 12, 2024. Data validation was performed in the EHR records for fifty patients. The study followed the RECORD-PE guidelines for reporting pharmacoepidemiological studies using routinely collected health data (Supplemental Digital Content Table S6, http://links.lww.com/SLA/F393 for filled out RECORD-PE checklist, Supplemental Digital Content 1, http://links.lww.com/SLA/F392).^24^ This is an extension of the STROBE guideline.^25^ The primary outcome of this study was the prevalence of oral benzodiazepine prescriptions. Ethical approval was obtained from the local Institutional Review Board and informed consent was waived (MEC-2023-0142).

### Study Population

All adult patients (≥18 years) admitted to the surgical department at the Erasmus Medical Centre between September 8, 2018 and September 8, 2022 were eligible for inclusion. Patients were excluded if they had, before this study, explicitly requested their data not to be used for research purposes. Furthermore, patients with a hospital length of stay (LOS) shorter than 24 hours were excluded. Multiple admissions for individual patients were seen as separate admissions. The first admission of each patient was selected for a separate analysis (the “index” admission) to calculate the incidence of benzodiazepine administrations. All readmissions or subsequent admissions, regardless of timespan, were considered readmissions for the index admission analysis. The investigators only had access to data regarding readmission to surgical departments, and not for other specialties. Subdivision in subspecialties of the surgical department was as follows; general surgery (GEN), benign enterological surgery, trauma (TRAU) surgery, gastrointestinal and oncological surgery (GOS), vascular surgery (VAS), and transplantation and hepatopancreatobiliary surgery (THS).

### Baseline Demographics and Clinical Outcomes

Data were gathered on age, sex, hospital LOS, global duration and number of surgical procedures performed, surgical subspeciality, pain scores (NRS and/or VAS), Delirium Observation Scale scores, and (modified) Early Warnings Sign [(M)EWS] scores (used as a screening tool to identify critically ill patients in an early stage). The data extraction methodology could not automatically provide the data to determine when surgical procedures took place during admission. Consultations with geriatricians or psychiatrists during admission were extracted. Subgroups were stratified by age in blocks of 20 (<40, 40–59, 60–79, ≥80 years). In cases where patients had been admitted for different surgical subspecialties during the same admission, the last subspeciality was recorded as the main subspecialty. Maximum values of NRS/VAS, Delirium Observation Scale, and (M)EWS scores per admission were used in the analysis.

### Medication Data

The national registry for prescription medication, which is used by public and hospital pharmacies in the Netherlands, is incorporated in the EHR. This national registry is used for the registration of home medication and for medication reconciliation at admission. The EHR itself is robust for the use for assessing benzodiazepine use. The study hospital has a very strict medication protocol that requires a computerized prescription, followed by administration that needs to be confirmed by the nurse that administers the medication. This protocol is part of the quality system and is frequently audited. We cannot rule out incidental “missing” prescriptions, but this amount is very limited because there is a safety system in place: benzodiazepines in stock are counted daily (based on ordering) and compared with the administered benzodiazepines. The ward is notified by the pharmacy if there is a mismatch between the administered benzodiazepines according to the EHR and the stock at the ward. Intrahospital benzodiazepine use was defined as benzodiazepines administered between the start of admission until discharge and all benzodiazepines were dispensed by the hospital pharmacy. Data regarding benzodiazepine use after discharge were not accessible to the investigators and, therefore, not included in this study.

We collected data for oral prescriptions with the following ATC groups: N05BA, N05CD, and N05CF. Subsequently, we filtered the results for alprazolam, bromazepam, brotizolam, diazepam, flunitrazepam, flurazepam, loprazolam, lorazepam, lormetazepam, midazolam, nitrazepam, prazepam, oxazepam, temazepam, zolpidem, and zopiclone. An overview of used ATC codes can be found in Supplementary Table S1 (Supplemental Digital Content Table S1, http://links.lww.com/SLA/F393). We excluded prescriptions of intravenous, intranasal, buccal, and rectal benzodiazepines as these are generally used as anticonvulsive or anaesthesiological medications. In addition, we excluded midazolam orally, which is the first-choice premedication in the study hospital’s protocols, when this was clearly specified in the prescription (noted as special preoperative instruction). A distinction was made between prescriptions where the nurses did not register the administration to the patient (unfilled administration) and those where they did (completed administration). Benzodiazepine doses^26^ and Z-drug doses^27^ were converted to diazepam milligram equivalent (DME; Supplemental Digital Content Table S2, http://links.lww.com/SLA/F393). We also converted prescription data to defined daily dosages (DDDs) and defined daily doses of 10 mg diazepam equivalent (DDD-DME) to examine dosage regimens in the surgical subpopulation.^28,29^ Admissions of patients who received a benzodiazepine during hospitalization are hereafter referred to as benzodiazepine positive admissions, those that did not are referred to as benzodiazepine negative admissions. Admissions were considered “benzodiazepine naive” if the patient admitted was not registered as having used any oral benzodiazepines before this hospitalization, or past hospitalizations. To improve readability and limit the use of uncommon abbreviations, we will refer to the group of benzodiazepines and Z-related drugs simply as benzodiazepines.

### Statistical Analysis

Statistical analysis was performed using R studio (version 3.6.3 or higher), with packages “survival” and “gg2plot.”^30–32^ The analysis consisted of the following steps: firstly, the baseline demographics of the cohort were described. Continuous variables were described using the median (interquartile range). Categorical variables were described using number (percentage). Statistical differences between benzodiazepine positive and negative admissions for continuous and categorical variables were examined using Mann-Whitney *U* and χ^2^ tests, and Cohen D and Phi coefficients (φ), respectively. We conducted this baseline characteristic analysis for all admissions as well as the index admissions. Secondly, descriptive statistics were used to highlight differences in “time to first benzodiazepine taken,” and total DME used during admission. Mann-Whitney *U*, Kruskal-Wallis, and χ^2^ tests were used to examine outcomes. Thirdly, binary logistic regressions were used to examine factors associated with benzodiazepine use. In the first step, all factors were included in these regressions separately, estimating crude odds ratio (OR; and 95% CI). In addition, we included all characteristics in a multivariate model, estimating adjusted OR (OR; 95% CI). Again, we repeated this analysis in all admissions as well as the index admissions. For all admissions, we accounted for an additional factor “surgical readmission.” For the multivariate models, we checked for multicollinearity. As this does not exist for binary logistic regressions, we estimated the same model using linear regression and approximate variance inflation factors. Fourth, multivariate regression analysis was carried out to examine the influence of benzodiazepine use on surgical readmission. Covariates selected were based on literature, and included age, sex, LOS, number of surgeries performed during admission and surgical duration.^33,34^ Multicollinearity was assessed using the previously explained method. Univariate regression analysis was carried out before constructing the final model to evaluate model fit. We present the ORs (OR; 95% CI). All analyses were carried out for all admissions, unless otherwise specified. A *P* value <0.05 was considered statistically significant.

## RESULTS

An overview of baseline demographics of all admissions can be found in Table 1. In total, 14,928 admissions were included in the final analysis, representing 10,896 unique patients. Of the total, 8761 (58.7%) were males, with a median age of 62 (50–72). The median LOS in days was 4.5 (2.0–9.2) and a surgical procedure was performed in 10,994 out of 14,928 (73.3%) admissions. The distribution of different subspecialties was as follows; THS 3254 (21.8%), GEN 3207 (21.5%), GOS 3156 (21.1%), TRAU surgery 2931 (19.6%), VAS 2185 (14.6%), and benign gastrointestinal surgery 195 (1.3%). Out of 14,928 (92.0%) admissions, 13,736 were considered benzodiazepines-naïve, meaning those patients did not use benzodiazepines before hospitalization. Data on dosage were missing for 693 (3.3%) tablets, which was mostly due to minor prescription and administrative errors.

**TABLE 1:** Patient Demographic and Prevalence of Benzodiazepine Use in All Admissions

	Total group, all admissions (n = 14928)		Benzodiazepine-positive admissions (n = 3219)		Benzodiazepine-negative admissions (n = 11709)			
	n (%)	Median (IQR)	n (%)	Median (IQR)	n (%)	Median (IQR)	*P*	Effect sizes
Age	—	62 (50–72)	—	63 (51–71)	—	62 (50–72)	0.86	0.015
Sex								
Female	6167 (41.3)	—	1455 (45.2)	—	4712 (40.2)	—	—	—
Male	8761 (58.69)	—	1764 (54.8)	—	6997 (59.8)	—	<0.001	0.041
Benzo naïve	13736 (92.02)	—	2497 (77.6)	—	11239 (96)	—	<0.001	0.279
LOS in days	—	4.5 (2.0–9.2)	—	8.7 (4.3–15.8)	—	3.9 (1.8–7.7)	<0.001	0.748
Surgical subspecialty								
GEN	3207 (21.5)	—	678 (21.1)	—	2529 (21.6)	—	—	—
GOS	3156 (21.1)	—	655 (20.3)	—	2501 (21.4)	—	—	—
BES	195 (1.3)	—	50 (1.6)	—	145 (1.2)	—	—	—
THS	3254 (21.8)	—	766 (23.8)	—	2488 (21.2)	—	—	—
TRAU	2931 (19.6)	—	604 (18.8)	—	2327 (19.9)	—	—	—
VAS	2185 (14.6)	—	466 (14.5)	—	1719 (14.7)	—	0.03	—
Surgeries performed	—	1.0 (0.0–1.0)	—	1.0 (1.0–1.0)	—	1.0 (0.0–1.0)	<0.001	0.354
Surgery performed y/n	10944 (73.3)	—	2505 (77.8)	—	8439 (72.1)	—	<0.001	0.053
Consultations								
PSY	184 (1.2)	—	109 (3.4)	—	75 (0.6)	—	<0.001	0.102
GER	105 (0.7)	—	39 (1.2)	—	66 (0.6)	—	<0.001	0.032
Surgical readmissions	1836 (12.3)	—	505 (15.7)	—	1331 (11.4)	—	<0.001	0.054
Maximum DOS score	—	1.0 (0.0–3.5)	—	2.0 (0.5–4.0)	—	1.0 (0.0–2.0)	<0.001	0.311
Maximum (M)EWS	—	2.0 (1.0–3.0)	—	3.0 (2.0–4.0)	—	2.0 (1.0–3.0)	<0.001	0.438
Maximum pain score	—	0.0 (0.0–0.0)	—	0.0 (0.0–0.0)	—	0.0 (0.0–0.0)	<0.001	0.191
Benzodiazepines use								
Intake	3219 (21.6)	—	—	—	—	—	—	—
Prescription	3496 (23.4)	—	—	—	—	—	—	—

Statistics used: 1. Mann-Whitney *U*, 2. χ²-test, 3. Cohen D, 4. Phi coefficient.

BES indicates benign enterological surgery; DOS, Delirium Observation Scale; GER, geriatrics; PSY, psychiatry.

Of all admissions, 3,219 (21.6%) were administered a benzodiazepine at least once during hospitalization, representing the prevalence of benzodiazepine administrations. Using the index admissions to calculate the incidence, 2299 out of 10,896 patients received at least one benzodiazepine (21.1%). An overview of results for index admissions can be found in Supplemental Table S3 (Supplemental Digital Content Table S3, http://links.lww.com/SLA/F393).

**FIGURE 1:**
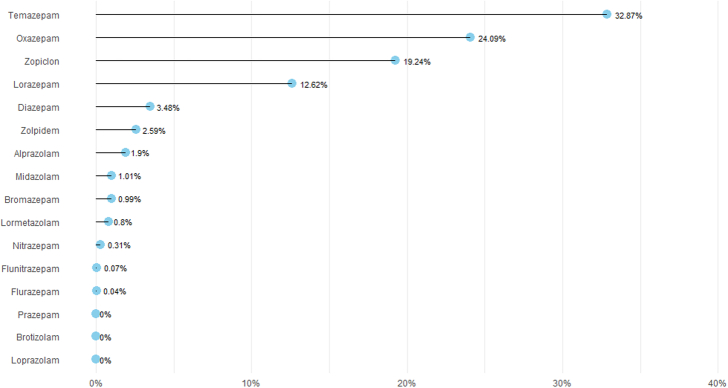
Lollipop chart of proportions of all benzodiazepines administered.

A total of 20,949 single doses of benzodiazepine tablets were administered. The median number of single administrations per patient in the benzodiazepine-positive group was 3 (1–7). The most frequently administered benzodiazepine was temazepam (32.9%), followed by oxazepam (24.1%), zopiclone (19.2%), and lorazepam (12.6%). Results can be found in Figure 1 and Supplemental Table S4 (Supplemental Digital Content Table S4, http://links.lww.com/SLA/F393).

A total of 12,891 defined daily doses of 10 mg of diazepam equivalent (DDD-DME) were administered to 3219 patients during the 4-year study period. A benzodiazepine-positive admission, on average, received 4 defined daily doses of 10 mg diazepam equivalents. After conversion of the total amount of benzodiazepines used during the study period to DDDs, the most consumed benzodiazepines were temazepam (4032 DDD), zopiclone (3991 DDD), lorazepam (1178 DDD), and oxazepam (968 DDD). The average dose per single administration of the 5 most common benzodiazepines was 11.9 mg for temazepam, 7.8 mg for zopiclone, 9.8 mg for oxazepam, 1.2 mg for lorazepam, and 6.6 mg for diazepam (full results can be found in Supplemental Digital Content table S5, http://links.lww.com/SLA/F393).

The result of the subgroup analysis with corresponding effect sizes can be found in Table 1. Age was not significantly different between benzodiazepine-positive and benzodiazepine-negative admissions (63 vs 62 years, *P* = 0.86). For admissions of male patients, a total of 20.1% were benzodiazepine positive, for female patients, this corresponded to 23.6% (1764 out of 8761 vs 1455 out of 6167, *P* < 0.001).

A significant difference was found in the length of the hospital stay (LOS) of benzodiazepine-positive admissions and benzodiazepine-negative admissions (8.7 vs 3.9 days; *P* < 0.001). Furthermore, a significant difference was observed in (M)EWS (3.0 vs 2.0; *P* < 0.001). Benzodiazepine naivety differed between both groups, with benzodiazepine-positive admissions having a lower proportion of benzodiazepine-naive patients. (77.6% vs 96.0%, *P* < 0.001).

**FIGURE 2:**
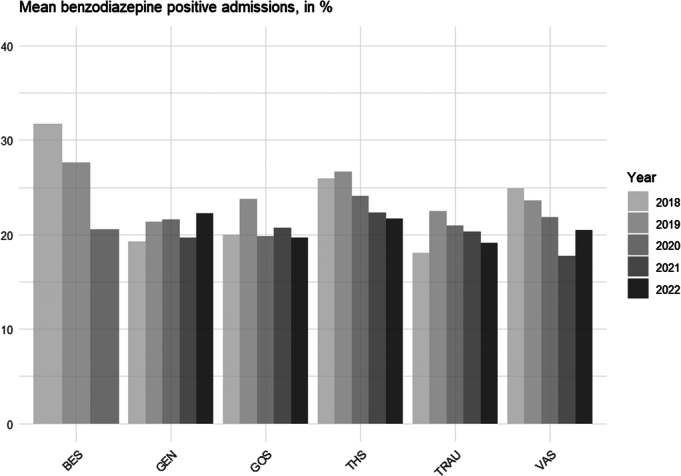
During the study period, the BES department merged with GOS. Therefore, no admissions for BES were included from 2020 onwards. BES indicates benign enterological surgery.

Furthermore, there seems to be a decreasing trend in prevalence of benzodiazepine use over time and subspeciality. An overview of prevalence per year and subspeciality can be found in Figure 2. Significant differences were found between year (*P* = 0.041), month (*P* = 0.036), and subspeciality (*P* = 0.027).

Median time to first benzodiazepine administration was 1 (0–5) day. Male patients received their first benzodiazepine at a median of 2 (0–6) days versus 1 (0–4) days for female patients (*P* < 0.001). Furthermore, a significant difference was found in “time to first benzodiazepine” for different subspecialties. GOS administered the first benzodiazepine at a median of 2 (0–6) days, which is significantly later compared with other subspecialties (*P* = 0.005). No significant differences were found for “time to first benzodiazepine” for months and years. See Supplemental Figure S1 (Supplemental Digital Content Fig.e S1, http://links.lww.com/SLA/F393) for a graphical representation of the timing of benzodiazepine distributions in the first 30 days of admission.

The subspecialties that administered the highest median total DME dose of benzodiazepines were TRAU (15 mg), GOS (15 mg), and GEN (15 mg). THS (10 mg) and VAS (10 mg) administered the lowest total DME dose (*P* < 0.001). From 2019, a decreasing trend was observed in the mean total DME prescribed until 2021. However, 2022 showed an increase in total DME administered.

**FIGURE 3:**
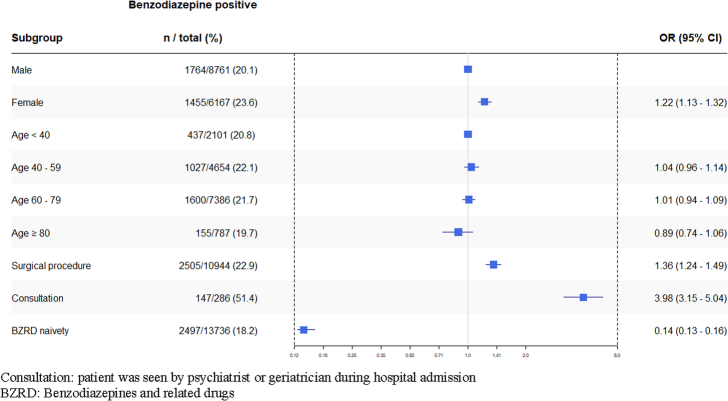
Forest plot of crude OR and 95% CI in all admissions. Consultation: patient was seen by psychiatrist or geriatrician during hospital admission. BZRD indicates benzodiazepines and related drugs.

**FIGURE 4:**
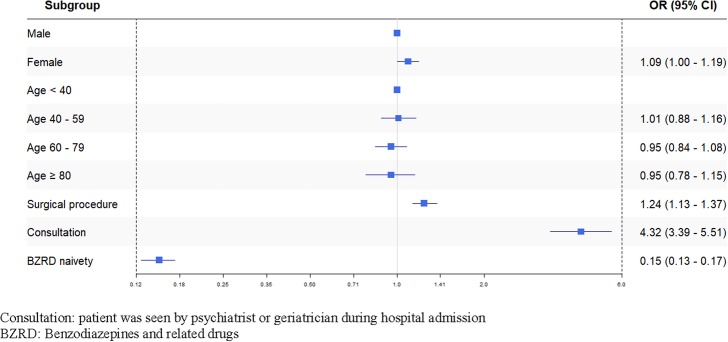
Forest plot of adjusted OR and 95% CI in all admissions. Consultation: patient was seen by psychiatrist or geriatrician during hospital admission. BZRD indicates benzodiazepines and related drugs.

The ORs of the univariate and multivariate logistic regressions of benzodiazepine use during hospitalization in all admissions can be found in Figures 3 and 4. Female patients had a higher likelihood of benzodiazepine administration during hospitalization (OR: 1.22; 95% CI: 1.13–1.32). Also, a consultation from a psychiatrist or geriatrist (OR: 3.98; 95% CI: 3.15–5.04) and having a surgical procedure during admission (OR: 1.36; 95% CI: 1.24–1.49) were positively associated with benzodiazepine administration. A priori benzodiazepine naivety was associated with a lower likelihood of benzodiazepine use during hospitalization (OR: 0.14; 95% CI: 0.13–0.16). In the multivariate analyses, similar results were found for sex (female; OR: 1.09; 95% CI: 1.00–1.19), and having a surgical procedure (OR: 1.24; 95% CI: 1.13–1.37). The same analysis was repeated for the index admissions, which can be found in Supplemental Figure S2 (Supplemental Digital Content Fig. S2, http://links.lww.com/SLA/F393) and Supplemental Figure S3 (Supplemental Digital Content Fig. S3, http://links.lww.com/SLA/F393). No large differences between populations were observed.

The concomitance between intrahospital benzodiazepine use, patient factors, and surgical readmission within 30 days was investigated. A significant association was found in univariate analysis, using only benzodiazepine intake and surgical readmission (OR: 1.45; 95% CI: 1.30–1.62). The association between benzodiazepine use and surgical readmission was further investigated using multivariate analysis. Here, the association persisted (OR: 1.37; 95% CI: 1.22–1.54), indicating that benzodiazepine use is a predictor for surgical readmission in this cohort. After adjusting for possible confounding factors, the multivariate model showed improved fit compared with the univariate model (Akaike information criterion for the multivariate model: 10,982 versus Akaike information criterion for the univariate model: 11,094). Also, we found no evidence for multicollinearity (variance inflation factor <1.5).

## DISCUSSION

In this retrospective cohort study, we explored the patterns and trends in oral benzodiazepine administration in surgical patients in a large tertiary hospital. Simple descriptive statistics showed that in 21.6% of admissions to a surgical ward, oral benzodiazepines were administered to patients. The most frequently prescribed benzodiazepines were temazepam, oxazepam, and zopiclone, respectively.

The comparison of our results with similar studies is difficult due to heterogeneity in the study population, medication assessed, and methodology. This study was performed in a well-funded and well-equipped university medical centre in North-Western Europe, limiting generalizability. Yet, the prevalence of benzodiazepine use of 21.6% in our study is not an outlier compared with previous studies. Benzodiazepine use in the general hospital population in different point-prevalence studies ranged from 10% to 30%.^35–38^ Benzodiazepine prevalence in cohort studies of different groups of hospitalized patients ranged from 26.7% to 48.3%.^22,39,40^ According to the Dutch Foundation for Pharmaceutical Metrics (SFK), about 10% of the Dutch adult population retrieved benzodiazepines from pharmacies in the Netherlands during the corresponding study period.^17^ Comparing our results to the general population, the prevalence of intrahospital use of benzodiazepines is higher. However, benzodiazepine prescription and use also reflect the culture of a society, a hospital, or a ward—depending on the level studied. We, therefore, encourage researchers and hospitals throughout the world to replicate our study and report benzodiazepine use.

In this cohort, female patients were more likely to be prescribed benzodiazepines and also received the first benzodiazepine earlier during their hospital stay than male patients. This is in line with findings from similar studies, which have found a higher frequency of prescriptions for hypnotic drugs among women compared with men.^16,18,20,36,41,42^ It was not possible to retrieve the specific indications for individual administrations of benzodiazepines. However, two of the most common indications for oral benzodiazepines are the treatment of sleep disturbance and anxiety.^36^ Preliminary data from a retrospective cohort in the same hospital, partially overlapping departments and study period, revealed that women more often report sleep disturbances and anxiety, which are commonly treated with benzodiazepines. Another reason for this difference might be a social bias, a study from Taiwan in patients with a psychiatric disorder suggests that male prescribers are more likely to prescribe benzodiazepines to females.^43^ However, this does not explain the second observed sex difference, that females are given benzodiazepines earlier during hospital stay. We hypothesize that female patients report symptoms of anxiety and sleep disturbance earlier than male patients (to the mostly female nursing staff), leading to earlier benzodiazepine use.

Furthermore, our results indicate that benzodiazepine use is associated with 30-day surgical readmission. However, due to limited access to possible confounders such as prior medical history and complexity of surgery, these results should be interpreted with caution. For similar reasons, we decided against reporting on possible correlations between benzodiazepine use and mortality in our study. Our study was not designed to evaluate mortality, and our results would suffer from a lack of access to comorbidity data and have indication bias. It is important to note that larger cohort studies designed specifically to elucidate a possible association between benzodiazepine use and mortality are inconsistent and have been inconclusive.^44–48^

Lastly, the study period includes the onset of the COVID-19 pandemic, which had a variety of effects on patients and the health care system as a whole, and possibly on benzodiazepine use.^49,50^ Any potential confounding due to “COVID-19 effects” can therefore not be ruled out, although we did not see large differences between benzodiazepine use in COVID years and non-COVID years.

To summarize, we believe that the proportion of benzodiazepine usage in this study population is high. In light of the addictive properties of benzodiazepines and the increasing rate of concomitant use of benzodiazepines and opioids, efforts to reduce benzodiazepine use are warranted. As non-pharmacological alternatives such as music have emerged for the treatment of sleep disturbance and anxiety,^51,52^ we believe efforts should be made to decrease the rate of intrahospital benzodiazepine administrations.

### Limitations

This study has a number of limitations. Firstly, the retrospective and observational nature of this cohort study prevents establishing causal relations between observations. Secondly, the EHR from which the data were collected does not log indications for drug prescriptions, and thus we were unable to ascertain the specific indication for the benzodiazepine administration. Moreover, because a number of benzodiazepines, such as lorazepam and oxazepam, are registered treatments for both sleep disturbance and anxiety, we cannot reliably deduce for which indication it was given. Thirdly, we did not have access to individual diagnoses or past medical history of patients, limiting our ability to control for potential confounders such as cancer diagnoses or emergency surgical procedures. Lastly, although the DME metric is often used in literature, it is less evidence based and has underwent less validation than, for example, the morphine milligram equivalence.^29^

## CONCLUSIONS

Approximately one-fifth of all patients (21.6%) admitted to a surgical department are administered oral benzodiazepines, with a focus on treating sleep disturbances and anxiety. Female patients are treated with benzodiazepines earlier and more frequently than male patients. This study reports associations between benzodiazepine usage and increased rate of early readmission. Given the potential addictive properties and harmful side effects of benzodiazepines, the authors recommend non-pharmacological alternatives for the treatment of anxiety and sleep disturbances.

## Supplementary Material
